# PlantForm-Grown Shoots of *Rhaponticum carthamoides* (Willd.) Iljin as a Source of Caffeoylquinic Acid Derivatives and Antioxidant Potential of Shoot Extract

**DOI:** 10.3390/molecules30244724

**Published:** 2025-12-10

**Authors:** Ewa Skała, Agnieszka Kicel

**Affiliations:** 1 Department of Biology and Pharmaceutical Botany, Medical University of Lodz, Muszynskiego 1, 90-151 Lodz, Poland; 2 Department of Pharmacognosy, Medical University of Lodz, Muszynskiego 1, 90-151 Lodz, Poland; agnieszka.kicel@umed.lodz.pl

**Keywords:** temporary immersion system, caffeoylquinic acid derivatives, hydroxyl radical scavenging assay, hydrogen peroxide reduction assay, superoxide anion scavenging assay

## Abstract

*Rhaponticum carthamoides* (Maral root) is an important medicinal plant species in Siberia and Kazakhstan. The aim of this study was to evaluate the effect of cultivation time (three or five weeks) and immersion frequency (i.e., every 1.5 h, 3 h, or 6 h) on the growth of *R. carthamoides* shoots in a temporary immersion PlantForm bioreactor; it examines the effect of cultivation on the accumulation and productivity of caffeoylquinic acid derivatives, shoot biomass and propagation. Both growth time and frequency of immersion affected the increase in biomass and phenolic compound production. The highest dry weight (9.35 g/L) was observed for shoots grown for five weeks and immersed every 1.5 h; optimal synthesis (4.5 mg/g DW) and productivity of caffeoylquinic acid derivatives (16 mg per bioreactor) was noted following immersion every three hours. The main class of synthesized compounds was mono-caffeoylquinic acid derivatives (3.3 mg/g DW; 11.85 mg/bioreactor) with chlorogenic acid predominating (2.9 mg/g DW; 10.4 mg/bioreactor), as determined by HPLC-PDA. The antioxidant activity of bioreactor-grown shoot extract was assessed in vitro using three cell-free assay systems: hydroxyl radical (OH^•^) scavenging, hydrogen peroxide (H_2_O_2_) reduction, and superoxide anion (O_2_^•−^) scavenging. Our findings indicate that the PlantForm bioreactor can be successfully used to grow *R. carthamoides* shoots and produce valuable caffeoylquinic acid derivatives.

## 1. Introduction

*Rhaponticum carthamoides* (Willd.) Iljin, commonly known as Maral root, is a species belonging to Asteraceae family. It is an endemic plant that grows naturally in Siberia, Mongolia or Kazakhstan [1,2,3,4]. The roots and rhizomes are well known in the traditional medicine of Siberia. In addition, *Rhapontici carthamoidis rhizomata cum radicibus* have been monographed in the State Pharmacopoeia of Russia 14th edition as a tonic and adaptogen agent [4]. The species is characterized by a broad range of pharmacological effects [1,4], resulting from the presence of various bioactive compounds [1,3,5,6]. One particularly important class of compounds comprises the caffeoylquinic acid derivatives (CQAs), which exhibit diverse biological activities [1,7,8].

As a consequence of the over-exploitation of *R. carthamoides* in its natural habitat, the plant species is included in the Red List of the Russian Federation and regional Red List (the Red Book of Kazakhstan) [9,10]. Its propagation is also complicated by the slow growth of seedlings. *R. carthamoides* can also be propagated from rhizomes, but this requires plants that are three to four years old [10,11,12].

However, recent advances in in vitro micropropagation have made it possible to protect endangered medicinal plant species and support their multiplication, regardless of climatic conditions and seasons. In addition, the plants obtained by clonal mass propagation are characterized by high quality and are genetically identical to the parent plant. Biotechnological techniques also facilitate the enhanced production and extraction of valuable secondary metabolites without damaging plantations or the natural resources of plants. The plants can be multiplied on a large scale in liquid media in different types of bioreactors. Among these approaches, bioreactors have become favored for micropropagation, especially due to reduced production expenses and the potential to scale up the system [13]. One in vitro plant propagation technique is the temporary immersion system (TIS), in which plant tissues are cyclically immersed in a liquid medium, interspersed with periods of aeration and drying. The selection of these intervals has a significant influence on plant material growth in the TIS system [14,15,16,17,18].

Despite its pharmacological and ecological importance, *R. carthamoides* has not been widely propagated under in vitro conditions, and only a few studies have addressed the development of effective biotechnological methods for its propagation and metabolite production [10,19,20,21,22]. Given the urgent need to conserve this valuable species and to provide sustainable sources of bioactive compounds, the aim of the present study was to identify the optimal conditions for in vitro shoot culture of *R. carthamoides* using a temporary immersion system, PlantForm bioreactor; more specifically, it examines the effect of two parameters on the efficiency of shoot proliferation and the accumulation of CQAs, i.e., its pharmacologically active constituents. The analysis focusses on the influence of culture duration (three or five weeks) and immersion frequency (every 1.5, 3, and 6 h).

This study constitutes the first comprehensive evaluation of *R. carthamoides* shoot culture performance in the TIS bioreactor system and provides new insights into the influence of cultivation parameters on plant growth and metabolite accumulation.

## 2. Results

### 2.1. Caffeoylquinic Acid Derivative and Flavonoid Production

**Figure 1:**
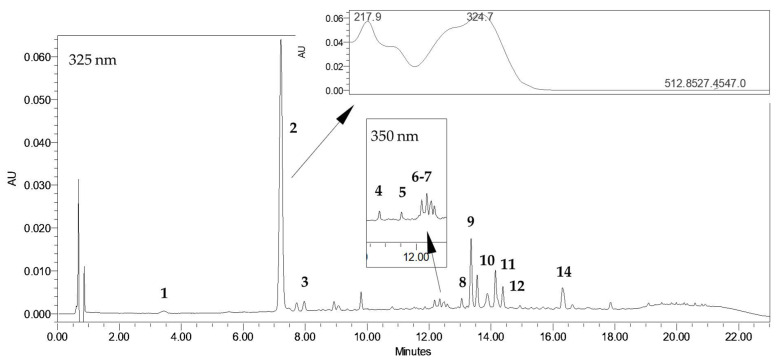
HPLC-UV chromatogram (λ = 325 nm) of extract of PlantForm-grown shoots of *R. carthamoides* immersed every 3 h, cultured for 5 weeks. 3-*O*-caffeoylquinic acid (3-CQA) (**1**); 5-*O*-caffeoylquinic acid (5-CQA, chlorogenic acid) (**2**); 4-*O*-caffeoylquinic acid (4-CQA) (**3**); quercetagetin hexoside (**4**); quercetin hexoside (**5**); luteolin hexoside (**6**); patuletin hexoside (**7**); 3,4-*O*-dicaffeoylquinic acid (3,4-diCQA) (**8**); 3,5-*O*-dicaffeoylquinic acid (3,5-diCQA) (**9**); 1,5-*O*-dicaffeoylquinic acid (1,5-diCQA) (**10**); 4,5-*O*-dicaffeoylquinic acid (4,5-diCQA) (**11**); dicaffeoylquinic acid (di-CQA) (**12**); tricaffeoylquinic acid 2 (tri-CQA 2) (**14**).

*R. carthamoides* shoots grown in PlantForm bioreactor are a rich source of CQAs (2.17–4.46 mg/g dry weight (DW)) and flavonoids (0.13–0.47 mg/g DW) (Table 1). These specialized metabolites were detected using a UPLC-PDA-ESI-MS^3^ method, as described in earlier studies [22,23]. Figure 1 presents a representative UPLC-UV chromatogram of aqueous methanol extract from *R. carthamoides* bioreactor-grown shoots. The extracts were found to contain the following constituents: three mono-CQAs: 3-*O*-caffeoylquinic acid (3-CQA) (**1**); 5-*O*-caffeoylquinic acid (5-CQA, chlorogenic acid) (**2**); 4-*O*-caffeoylquinic acid (4-CQA) (**3**)); five di-CQAs: 3,4-*O*-dicaffeoylquinic acid (3,4-diCQA) (**8**); 3,5-*O*-dicaffeoylquinic acid (3,5-diCQA) (**9**); 1,5-*O*-dicaffeoylquinic acid (1,5-diCQA) (**10**); 4,5-*O*-dicaffeoylquinic acid (4,5-diCQA) (**11**); dicaffeoylquinic acid (di-CQA) (**12**); two tri-CQAs: tricaffeoylquinic acid 1 (tri-CQA 1) (**13**); tricaffeoylquinic acid 2 (tri-CQA 2) (**14**), and flavonoids: quercetagetin hexoside (**4**); quercetin hexoside (**5**); luteolin hexoside (**6**); and patuletin hexoside (**7**).

The main group of compounds were mono-CQA derivatives (1.27–3.27 mg/g DW), which accounted for over 50% of all identified CQAs, regardless of the culture conditions (Table 1).

**Figure 2:**
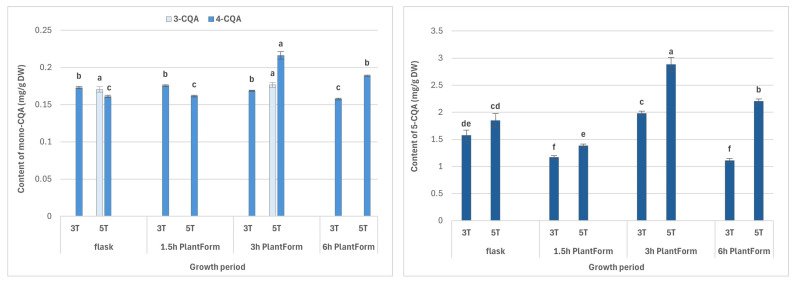

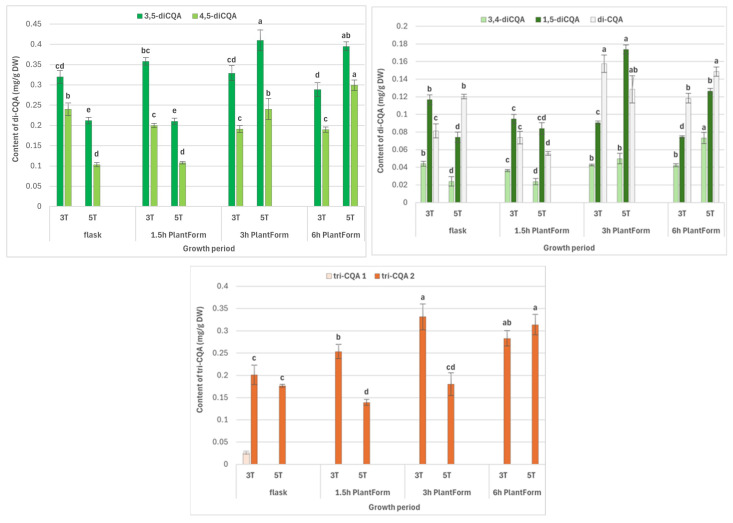
Effect of cultivation time (3 and 5 weeks) and immersion frequency (every 1.5 h, 3 h, and 6 h) on the content of individual mono-, di-, and tri-CQAs (mg/g DW) in PlantForm-grown shoots of *R. carthamoides*. The results are mean values ± SD. No difference (*p* < 0.05) is present between means for the same metabolite marked with the same letter. 3T—3 weeks of growth; 5T—5 weeks of growth; the compound codes correspond to those applied below the chromatogram in Figure 1; tricaffeoylquinic acid 1 (tri-CQA 1) (t_R_ = 15.6 min).

The length of cultivation (i.e., three or five weeks) and immersion frequency (every 1.5 h, 3 h or 6 h) significantly affected the content of individual specialized metabolite (Figure 2). For some phenolic acids, higher levels were noted for longer culture periods. The highest level of CQAs, i.e., the sum of all identified CQAs (4.46 mg/g DW), was observed after five weeks, in shoots immersed every three hours, followed by those immersed every six hours (3.75 mg/g DW). The lowest level was noted in shoots immersed 16 times per day (2.17 mg/g DW) (Table 1).

The most abundant compound was 5-CQA (chlorogenic acid; **2**), whose level ranged from 1.11 mg/g DW to 2.88 mg/g DW, depending on the culture conditions; its highest content was detected in five-week-old shoots immersed eight times per day (Figure 2). Other dominant caffeoylquinic acid derivatives were 3,5-diCQAs (0.21–0.41 mg/g DW), 4,5-diCQA (0.11–0.30 mg/g DW), and tri-CQA 2 (0.14–0.33 mg/g DW) (Figure 2).

The maximum levels of di-CQA derivatives were found after five weeks (Table 1). For 3,5-diCQA, no significant change in content was noted for immersion every 3 or 6 h; for 4,5-diCQA, the highest level was recorded following immersion every six hours (Figure 2). For tri-CQA 2, longer intervals between shoot immersion and shorter period growth increased biosynthesis; however, no significant differences in content were observed for immersion every six hours (Figure 2).

The shoots cultivated under optimal conditions in the PlantForm bioreactor exhibited higher metabolite content than those grown in stationary flasks in liquid medium (Table 1 and Figure 2). Chlorogenic acid was increased 1.5-fold, and the levels of 4,5-diCQA and 3,5-diCQA were increased around two-fold.

For flavonoids, optimal accumulation was observed after five weeks in shoot culture with a three-hour immersion interval (0.47 mg/g DW). Similar flavonoid contents (0.4 mg/g DW) were obtained in shoot cultures grown in the liquid medium, in the stationary flasks for three weeks, and PlantForm-grown shoots immersed every six hours (Table 1).

### 2.2. Shoot Micropropagation

**Figure 3:**
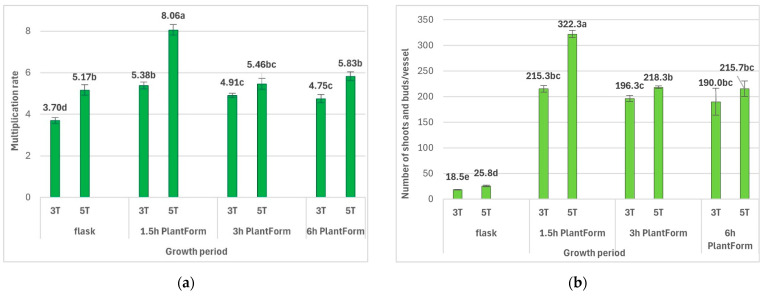
Effect of the cultivation time (3 and 5 weeks) and immersion frequency (every 1.5 h, 3 h, and 6 h) on the growth of PlantForm-grown shoots of *R. carthamoides*: (**a**) multiplication rate, (**b**) number shoots and buds per one flask or bioreactor. The results are mean values ± SE. There is no difference (*p* < 0.05) among the means with the same letter. 3T—3 weeks of growth; 5T—5 weeks of growth.

**Figure 4:**
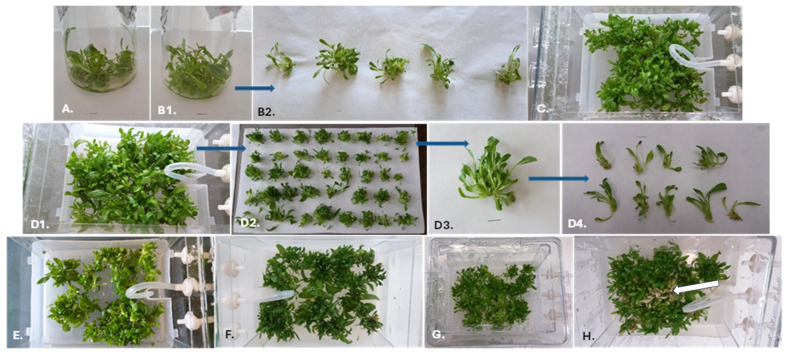
*R. carthamoides* shoots cultured in: stationary flask for 3 (**A**) and 5 weeks (**B1**,**B2**); PlantForm bioreactor immersed every 1.5 h for 3 (**C**) and 5 weeks (**D1**–**D4**); PlantForm bioreactor immersed every 3 h for 3 (**E**) and 5 weeks (**F**); PlantForm bioreactor immersed every 6 h for 3 (**G**) and 5 weeks (**H**). Bar = 1 cm. The white arrow indicates the shoots displaying necrosis.

The duration of culture and immersion frequency also significantly influenced the number of new buds and shoots of *R. carthamoides*. Firstly, the shoots required a more extended cultivation period. The optimal multiplication rate of *R. carthamoides* shoots (8.1 shoots and buds per explant) was observed for 1.5 h of immersion and five weeks of growth (Figure 3 and Figure 4). Extending the time between immersions, i.e., 4 or 8 times per day, reduced the number of shoots and buds to 5.5 and 5.8, respectively. In addition, the frequency of immersion, i.e., every three or six hours, did not have a statistically significant effect on shoot multiplication (Figure 3). The multiplication rate for *R. carthamoides* shoots cultured in a stationary flask was lower, amounting to 5.2 shoots and buds per explant. Regarding the number of multiplied structures during a single cycle (after five weeks), 216–322 new shoots and buds were produced from one bioreactor (Figure 3), compared to only 26 new shoots and buds from a single flask (Figure 3).

Our findings indicate that *R. carthamoides* shoots were well adapted to growth in the PlantForm bioreactor. The TIS system positively influenced the quality of the propagated plant material. The length of shoots after five weeks of culture ranged from 2.06 cm to 2.46 cm, depending on immersion conditions (Table 2). A higher percentage of buds (max. 25.5%) compared to all newly formed structures was noted after three weeks compared to five weeks (max. 9.8%); this was dependent on the culture conditions (Table 2), with the lowest percentage recorded for shoots immersed four times a day. In addition, after five weeks, only 4.1–9.6% shoots exhibited hyperhydricity symptoms (Table 2). The shoots immersed four times a day showed the highest percentage of hyperhydricity. Hence, the use of liquid medium and the TIS system support *R. carthamoides* shoot cultivation. The plant materials were good quality and could be used for further phytochemical analysis.

### 2.3. Biomass Enhancement

**Figure 5:**
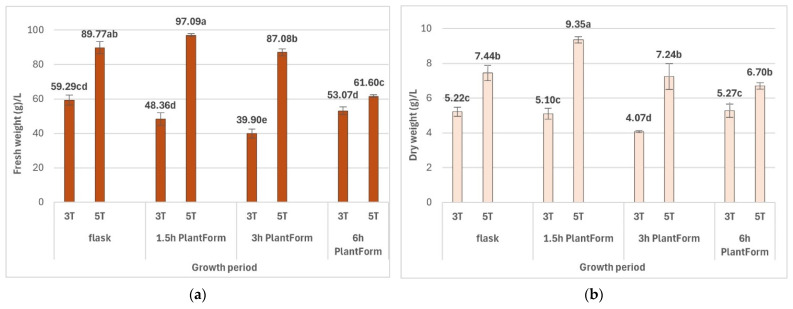
Effect of cultivation time (3 and 5 weeks) and immersion frequency (every 1.5 h, 3 h, and 6 h) on (**a**) fresh weight (g/L) and (**b**) dry weight (g/L) of *R. carthamoides* shoots. The results are mean values ± SE. Means marked with the same letter indicate no significant difference (*p* < 0.05). 3T—3 weeks of growth; 5T—5 weeks of growth.

The high multiplication efficiency of *R. carthamoides* shoots in the TIS system resulted in significant biomass growth. It was also observed that the time of the culture affected the biomass of shoots and depending on the culture conditions. The fresh (FW) and dry weight (DW) of shoots cultured for five weeks were significantly higher compared to those cultured for three weeks. The higher biomass yields were obtained when *R. carthamoides* shoots were immersed 16 times per day, *viz.* 97.09 g/L FW and 9.35 g/L DW (Figure 5); this was due to a higher number of shoots compared to the other culture conditions.

The lowest fresh biomass increase (61.60 g/L) was noted for shoots immersed four times per day (Figure 5). After five weeks of cultivation, the shoots covered the bottom of the culture vessel; high shoot density prevented coverage with the medium, and some (approx. 10%) were brown, dried out with signs of necrosis (Figure 4H). It is possible that this could be avoided by extending the immersion time of the plant material. Indeed, a preliminary study found that extending the interval between immersions to twice a day resulted in most of the shoots dying after five weeks; this experimental variant was excluded from this study.

### 2.4. Productivity of Caffeoylquinic Acid Derivatives

**Figure 6:**
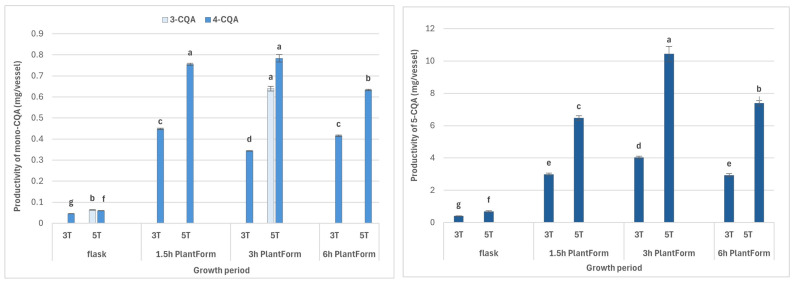

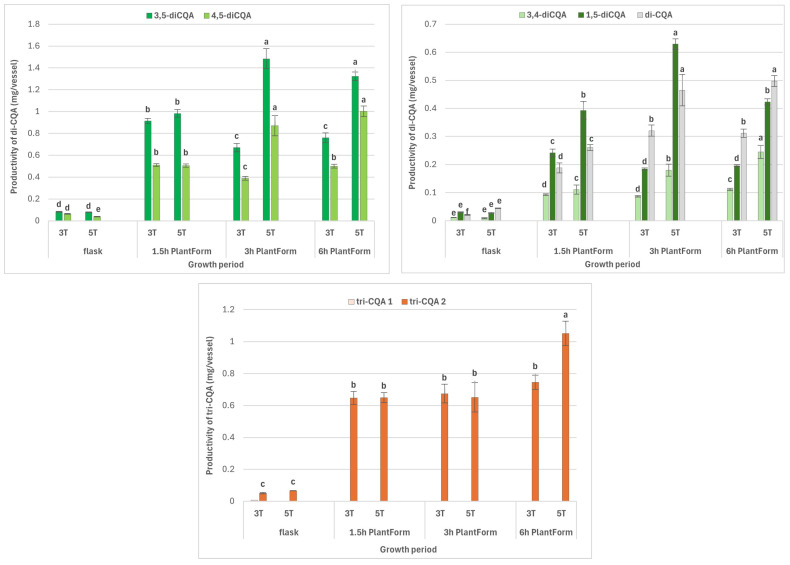
Effect of cultivation time (3 and 5 weeks) and immersion frequency (every 1.5 h, 3 h, and 6 h) on the productivity of individual mono-, di-, and tri-CQAs (mg/vessel in one cycle ± SE) in PlantForm-grown shoots of *R. carthamoides*. The results are mean values ± SD. Means for the same metabolite marked with the same letter indicate no significant difference (*p* < 0.05). 3T—3 weeks of growth; 5T—5 weeks of growth; the compound codes correspond to those applied below the chromatogram in Figure; tricaffeoylquinic acid 1 (tri-CQA 1) (t_R_ = 15.6 min).

A key parameter affecting efficient specialized metabolite accumulation in plant in vitro cultures is productivity, expressed as the yield of compounds from one litre of medium or one vessel in one growth cycle. Productivity reflects both an increase in dry biomass and the concentration of the specialized metabolites. Although the highest DW increase was achieved for *R. carthamoides* shoots cultured with a 1.5 h immersion interval, better metabolite content was achieved in shoots immersed every 3 h. Therefore, metabolite productivity per one cycle from a single bioreactor (or flask) was also presented. The highest total CQA productivity, 16.13 mg per one bioreactor cycle, was exhibited by shoots cultured for five weeks with immersion intervals every 3 h (Table 1); this value was approximately 1.5-times higher than that observed for other immersion frequencies. Moreover, it is worth emphasizing that during the same period, only 1 mg of CQAs could be obtained from a single flask. The following productivity values were obtained for the predominant CQAs: 10.43 mg for chlorogenic acid, 1.32–1.48 mg for 3,5-diCQA, and 0.87–1.00 mg for 4,5-diCQA (Figure 6).

### 2.5. Antioxidant Activity

**Figure 7:**
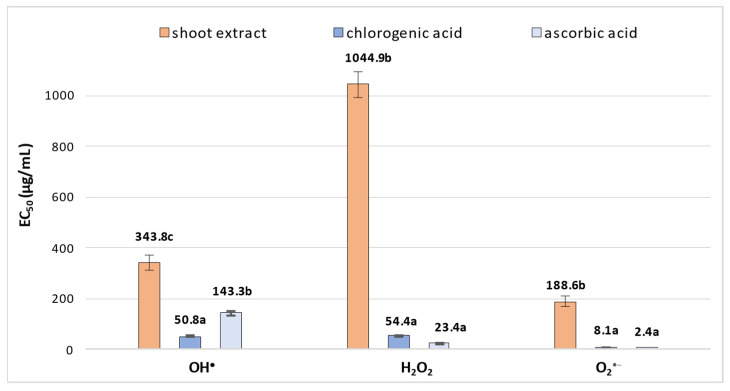
Assessment of antioxidant potential in PlantForm-grown shoots of *R. carthamoides*. The EC_50_ value reflects the concentration of the plant material or standards (µg/mL) needed to reduce the initial levels of OH^•^, H_2_O_2_, or O_2_^•−^ by 50%. Data are presented as mean values ± SE. Means followed by the same letter for the same assay do not differ significantly (*p* < 0.05).

As numerous studies have shown that CQAs exhibit strong antioxidant activity [7,8], the antioxidant potential of extract obtained from *R. carthamoides* shoots grown in the PlantForm bioreactor (which demonstrated the highest productivity of secondary metabolites) was also determined. The shoot extract was evaluated using three complementary in vitro antioxidant activity assays. The selected tests reflect some of the basic protective mechanisms of polyphenols under oxidative stress conditions, involving the inactivation of physiologically occurring reactive oxygen species (ROS): hydroxyl radical (OH^•^) scavenging, hydrogen peroxide (H_2_O_2_) reduction, and superoxide radical (O_2_^•−^) inactivation. *R. carthamoides* shoots exhibited a concentration-dependent ability to scavenge all target radicals, with overall antioxidant activity varying significantly: EC_50_ = 343.8 ± 30.5 µg/mL for the OH^•^ scavenging assay, EC_50_ = 1044.9 ± 52.2 µg/mL for H_2_O_2_ reduction, and EC_50_ = 188.6 ± 21.4 µg/mL for O_2_^•−^ inactivation (Figure 7). Variations in the quantitative phytochemical composition of the examined plant material also influence its biological activity. Due to the presence of CQAs in the selected *R. carthamoides* shoots, reaching concentrations of up to 4.5 mg/g DW (Table 1), the observed activity levels were significantly lower compared to those of the reference compound, chlorogenic acid. For chlorogenic acid, the results were EC_50_ = 50.8 ± 3.2 µg/mL for hydroxyl radical (OH^•^) scavenging, EC_50_ = 54.4 ± 0.03 µg/mL for hydrogen peroxide (H_2_O_2_) reduction, and EC_50_ = 8.1 ± 0.3 µg/mL for superoxide radical (O_2_^•−^) inactivation. *R. carthamoides* shoots exhibited the highest activity against OH^•^ and O_2_^•−^ radicals, with their activity against the hydroxyl radical being only half that of ascorbic acid (OH^•^ assay, EC_50_ = 143.3 ± 8.6 µg/mL) (Figure 7).

## 3. Discussion

Among the numerous plant species that serve as essential components of the human diet, many are sources of CQAs [7]. These compounds demonstrate a broad spectrum of bioactive properties beneficial to human health, including antioxidant effects. They primarily exert these effects through two mechanisms: by scavenging reactive oxygen species and by functioning as Michael acceptors that modulate the Keap1–Nrf2 signalling pathway [8]. The generation and activity of free radicals exert a profound influence on various physiological and pathological processes in the human body, with high levels resulting in the generation of oxidative stress. Consequently, CQAs, and other naturally occurring antioxidants, represent a promising avenue for the development of preventive and therapeutic strategies targeting diseases associated with radical overproduction [24].

As high levels of CQAs are present in *R. carthamoides* shoots, the present study focused on optimizing their cultivation in the TIS system, the PlantForm bioreactor, with the aim of scaling up shoot cultivation. Effective in vitro propagation aims to achieve rapid, high multiplication rate and biomass accumulation for the efficient synthesis of valuable specialized metabolites. The present study is the first to present a successful protocol for *R. carthamoides* propagation using PlantForm bioreactor (PlantForm AB, Hjarup, Sweden) and demonstrate the biosynthetic potential of the shoots obtained from the TIS system.

Firstly, it was found that cultivation time influenced *R. carthamoides* shoot growth (multiplication rate and biomass accumulation) and the accumulation of active ingredients. Secondly, immersion frequency was crucial for shoot growth. Most plant micropropagation protocols are based on solid media (typically gelled with agar); the shoots are usually grown in multiple small vessels (e.g., glass tubes, Magenta vessels), requiring the use of large numbers of them. Additionally, shoot growth on solid media incurs high agar costs.

However, a more effective option may be shoot cultivation in a liquid medium; the combination of liquid-agitated medium with blue light-emitting diodes (LED) has previously been found to offer optimal conditions for *R. carthamoides* shoot propagation and growth [22]. The liquid medium positively affected both shoot biomass increase and CQA accumulation. This could be related to the better availability of substrate components and tissue oxygenation [22]. Therefore, the next goal of our research was to increase production of CQAs in material obtained in vitro by scaling up the culture size and optimizing *R. carthamoides* shoot cultivation in a TIS system under blue LED light.

The TIS system has been successfully used for micropropagation of many plant species, including agroindustrial, ornamental or commercially important plants [17,25,26]. However, to achieve optimal propagation efficiency and plant biomass in the TIS system, it is necessary to optimise *inter alia* the immersion frequency and duration, volumes of medium and culture vessels, explant density, and ventilation [15,16,17,18]. Immersion intervals and duration influence nutrient uptake from the medium and water, which affects propagation efficiency and plant biomass increase [13]. Another advantage of the TIS system is that it prevents plant cultures from suffocation and excessive hydration: conventional liquid cultures in flasks continuously immersed in liquid medium during growth are often subject to hyperhydricity. In addition, this model minimizes operating costs by combining bioreactors into larger systems, and the liquid medium is easy to replace [13]. The TIS system also reduces mechanical stress compared to continuously agitated culture in flasks, which positively impacts the quality and growth of plants in vitro [27]. Also, in the TIS bioreactor, the medium periodically covers the explants under pump-induced overpressure, which keeps shear forces low.

The PlantForm bioreactor was developed for large-scale micropropagation in in vitro cultures [28]. It has been successfully used for the micropropagation of many medicinal plant species, including *Rhododendron tomentosum* [29], *Nasturtium officinale* [30], *Ruta corsica* [31], and *Ruta montana* [32]. In addition, TIS bioreactors have been utilized as a valuable tool for obtaining high yields of various specialized metabolites [25,29,30,31,32]. Periodic immersion and aeration of plant tissues during cultivation in the TIS systems influence nutrient availability as well as oxygen and gas exchange, and may be beneficial for increasing their biomass and producing secondary metabolites, which may ultimately reduce the costs of obtaining biologically active substances.

Our present findings confirm that cultivation time and the immersion frequency play key roles in biomass production and phenolic acid accumulation in *R. carthamoides* shoots grown in PlantForm bioreactors. The aqueous methanolic extracts were found to contain ten CQAs, representing mono- (**1**–**3**), di- (**8**–**12**), and tri-caffeoylquinic acid derivatives (**13**–**14**). The bioreactor conditions did not appear to influence the quality of the shoot extracts; however, only shoots grown in the PlantForm immersed every three hours biosynthesized 4-CQA, which was not identified in other conditions. Moreover, flavonoids, derivatives of quercetagetin, quercetin, luteolin, and patuletin, were also identified in all extracts. These compounds have been identified previously in *R. carthamoides* shoots grown in agar-solidified culture and liquid-agitated flasks [22]. The most common group of compounds detected in PlantForm-grown shoots was mono-CQA derivatives (1.27–3.27 mg/g DW), which represented over 50% of all identified CQAs. The main specialized metabolite was chlorogenic acid, regardless of the culture condition; this finding is similar to that noted in *R. carthamoides* shoots maintained in agar medium and liquid-agitated flasks [22].

An increase in culture period from three to five weeks improved the biomass and the multiplication rate of PlantForm-grown *R. carthamoides* shoots. The propagated material was good quality: the shoot length ranged from 2.1 cm to 2.5 cm. Over 90% of shoots exhibited normal morphology without symptoms of vitrification, and the percentage ratio of shoots to buds was high (90.2–99.9%). The highest DW yield after five weeks (9.35 g/L) was noted for *R. carthamoides* shoots immersed every 1.5 h. More frequent immersion causes the plant material to be covered with a thin layer of medium, which prevents drying and promotes better shoot growth. Extending the interval between shoot immersions to 3 h caused a decrease in shoot biomass to 7.2 g/L, and extending it to 6 h decreased biomass to 6.7 g/L. In addition, immersing the shoots every 12 h resulted in brown, dry shoots, and after five weeks, most had died (data not presented). Previous study has found immersion every 6 h to be the most effective frequency for the growth of *Gerbera jamesonii* shoots in a BIT^®^ bioreactor [33]. In addition, prolonging the time between immersion of *Myrtus communis* shoots in PlantForm bioreactor [13], and *Gardenia jasminoides* shoots in Rita bioreactor [27] from 4 h to 8 h also resulted in better shoot growth and higher shoot biomass.

The dry weight of *R. carthamoides* shoots cultured under optimal conditions in the PlantForm bioreactor (9.3 g/L) was 1.2-times greater than for shoots maintained in stationary flasks (7.4 g/L) and exceeded the value observed for shoots grown on agar-solidified medium (about 0.1 g/shoot) [22]. Similarly, *Chrysanthemum morifolium*, *Fragaria × ananassa*, and *Cnidium officinale* plantlets achieved higher dry biomass in a TIS system than in semi-solid and liquid culture [26].

Different immersion frequencies (every 1.5, 3, and 6 h) also affected the number of *R. carthamoides* shoots and buds per explant. Similar to the biomass of the shoots, immersion every 1.5 h was the most effective for both the proliferation rate (8.06) and the number of shoots per one bioreactor (322). The 1.5-fold lower number of shoots per explant was observed for shoots immersed every 3 h and 6 h. The frequency of immersion, 6 or 4 times per day, did not affect the value of *R. carthamoides* shoot multiplication rate (5.5 and 5.8, respectively). Rico et al. [34] observed similar results for the shoots of *Cannabis sativa* cultured in TIS Rita^®^ bioreactor. Increasing the frequency of immersion from once every 8 h to once every 4 h led to a similar multiplication coefficient. The immersion frequency also did not significantly affect the number of *Anthurium andreanum* cv. Rosa shoots [35]. On the other hand, literature data show that the frequency of explant immersion had a significant effect on the number of multiplied shoots of *Artemisia judaica* [36], *Stevia rebaudiana* [37,38], and *Hylocereus undatus* [39] in the Rita^®^ bioreactor. For example, for *Hylocereus undatus* shoots multiplied in TIS Ebb-and-Flow bioreactor (among different immersion frequencies (every 4, 8, 12, and 16 h)), the highest shoot multiplication was obtained for immersion of 2 min every 4 h [39]. For *Stevia rebaudiana* shoots, the best result (18 shoots per explant) was observed when they were cultured in Rita^®^ bioreactor at an immersion frequency of 2 min every 8 h [38]. The immersion frequency every 12 h, being a relatively long interval, may limit the availability of nutrients and other components in the culture medium, which caused the drying and death of PlantForm-grown *R. carthamoides* shoots. The adverse effect of such a long interval in immersing the plant material also negatively affected the proliferation of *Rubus idaeus* shoots [40].

Although *R. carthamoides* shoots growing in PlantForm bioreactor immersed 16 times per day achieved the highest biomass and the highest average number of new, multiplied structures after 5 weeks, the content of metabolites was the lowest. Frequent immersion tends to promote rapid biomass growth but may inhibit the signaling required for secondary metabolite synthesis, whereas less frequent immersion favours the accumulation of secondary metabolites at the expense of growth. Perhaps excessively long intervals between plant material immersions may act as a mild abiotic stress that triggers biosynthetic pathways for secondary metabolites and enhances gene expression involved in these pathways, but further study is required to explain this process. Similarly, *Gardenia jasmonoides* shoots cultured in Rita system, the highest biomass was achieved at 15 min every 8 h of immersion, while the maximum chlorogenic acid content occurred at 15 min every 12 h [27].

The culture time and immersion frequency had a significant effect on the CQAs production. A longer culture period improved phenolic production except tri-CQAs. A similar pattern in the production of specialized metabolites was noted during the cultivation of *R. carthamoides* transformed roots. The highest content of mono-CQAs was recorded in the later phase of the growth cycle, in contrast to the tri-CQAs level, which was highest before the culture reached the stationary growth phase [41].

The highest content of 5-CQA (2.88 mg/g DW) was detected in *R. carthamoides* shoots cultured for 5 weeks in PlantForm bioreactor and immersed 8 times per day. A similar level of chlorogenic acid was obtained in *R. carthamoides* shoots maintained for the same time in an agitated flask and agar medium, reaching a concentration of approximately 3 mg/g DW [22]. However, considering the biomass obtained from a single bioreactor, the productivity of chlorogenic acid in one cycle (after 5 weeks) was 10.4 mg. As reported Makowski et al. [42], the highest level of chlorogenic acid (0.3 mg/g DW) in *Pontechium maculatum* shoots was reached in shoots grown in PlantForm system; it was a 4- to 5-fold higher concentration than that found in shoots cultivated on solid medium (0.06 mg/g DW) and liquid agitated medium (0.07 mg/g DW). The larger capacity of PlantForm bioreactors compared to flasks allows for the simultaneous cultivation of significantly more plant explants (in the present study 40 vs. 5) and making them as ideal for plant propagation on a larger scale. In addition, the TIS bioreactor enables faster production of valuable secondary metabolites (shorter time of the shoot culture) compared to traditional soil-based cultivation, regardless of the season. The content of chlorogenic acid in the aerial parts of *R. carthamoides* plants cultivated in the field for 3 months was 6 mg/g DW [43]. The shoots grown in PlantForm bioreactor are capable to produce bioactive compounds effectively, with yields often exceeding or matching those of other in vitro culture methods. The TIS cultivation system also had a beneficial effect on the production of chlorogenic acid in *Pontechium maculatum* shoots [42], rosmarinic acid in *Salvia viridis* [44] and *Pontechium maculatum* shoots [42], ferulic acid and protocatechic acid in *Drosera peltata* shoots [45] or sinapinic acid in *Nasturtium officinale* shoots [30]. For example, the amount of rosmarinic acid increased to 31.6 mg/g DW in *Pontechium maculatum* shoots grown in PlantForm system. The content of this phenolic acid recorded in shoots grown in liquid agitated medium (7.4 mg/g DW) and on solid medium (4.9 mg/g DW) was 4- to 6-times lower [42]. Similarly, rosmarinic acid content in PlantForm-grown shoots of *Salvia viridis* was twice as high as in shoots grown on solid medium and 13-fold higher than in the above-ground parts of plants growing in the soil [44].

The productivity of the sum of all identified CQAs in *R. carthamoides* shoots cultured under optimal conditions in PlantForm bioreactor (16.1 mg/bioreactor; 32 mg/L) in one cycle (from single bioreactor), within 5 weeks was 1.5-time greater than in the stationary flasks (1.07 mg/flask; 21 mg/L) and 2-time lower than that growing in agitated flasks (68 mg/L) [22]. However, taking into account the number of vessels used and the space they occupy in the growth chamber, the TIS system is more advantageous. The productivity of the predominant CQAs, such as chlorogenic acid, 3,5-diCQA, and 4,5-diCQA, from *R. carthamoides* shoots grown in one PlantForm bioreactor was 10.4 mg, 1.5 mg, and 1 mg, respectively.

The antioxidant potential of *R. carthamoides* shoots grown in the PlantForm system was evaluated using three different in vitro assays. The shoots showed the strongest scavenging activity against hydroxyl (OH^•^) (EC_50_ = 344 µg/mL) and superoxide (O_2_^•−^) radicals (189 µg/mL), with their effectiveness against the hydroxyl radical being only about half that of the well-known antioxidant ascorbic acid (EC_50_ for OH^•^ scavenging = 143 µg/mL). The antioxidant potential of *R. carthamoides* leaf extracts was evaluated earlier using several assays, including the phosphomolybdenum method, β-carotene bleaching test, DPPH radical scavenging assay, and FRAP assay [46,47]. Methanolic and aqueous extracts demonstrated ferric ion-reducing capacity, although their activity was approximately 2.5 times lower than that of standard reference antioxidants. In the DPPH assay, the methanolic extract obtained by sonication exhibited antioxidant activity approximately 3.5-times higher (IC_50_ = 38 µg/mL) than that of BHT (IC_50_ = 141 µg/mL); however, its activity remained about 7-times lower than that of ascorbic acid (IC_50_ = 5.6 µg/mL) and 3-times lower than that of α-tocopherol (IC_50_ = 10.8 µg/mL) [46].

Chlorogenic acid, the principal marker compound identified in the *R. carthamoides* shoot extracts, also exhibited notable activity across all three assays, confirming its important contribution to the overall radical-scavenging potential of the plant samples. Structural features strongly determine the antioxidant properties of CQAs. Specifically, derivatives bearing two or three caffeoyl groups generally show higher antioxidant potency than mono-caffeoylquinic acids, due to the increased number of phenolic hydroxyl groups available for hydrogen atom transfer (HAT) or single-electron transfer (SET) reactions [48,49]. In addition, the position of caffeoyl substituents critically affects activity, with adjacent substituents (e.g., 3,4-*O*-di-CQA or 4,5-*O*-di-CQA) stabilizing phenoxyl radicals more efficiently and enhancing radical-scavenging potency [50]. Density functional theory (DFT) calculations, a quantum chemical approach that predicts molecular electronic structure and reactivity, have further confirmed that chlorogenic acid scavenges radicals via HAT and SET mechanisms, and that its local hydroxyl arrangement and conformational flexibility significantly influence its antioxidant efficacy in different environments [51]. Overall, although multi-caffeoylated CQAs may contribute to antioxidant activity, in *R. carthamoides* shoot extracts, chlorogenic acid is expected to exert the dominant influence due to its several times higher concentration relative to di- and tri-CQAs. Therefore, it is more likely that the minor CQAs act synergistically with chlorogenic acid than serve as primary contributors to the activity profile.

## 4. Materials and Methods

### 4.1. Plant Materials

Aseptically grown shoots were obtained for the present study [22]. The shoots were taken from fragments of stems with an axillary bud and were multiplied on Murashige and Skoog (MS) [52] agar (0.7%) (Duchefa Biochemie B.V., Haarlem, The Netherlands) medium supplemented with 6-benzyladenine (BA) (0.5 mg/L) and indole-3-acetic acid (IAA) (0.1 mg/L) (Duchefa Biochemie B.V., Haarlem, The Netherlands). The shoots were cultured in a growth chamber at 26 ± 2 °C under blue LED light conditions (460 nm) at Photosynthetic Photon Flux Density (PPFD) of 35 μmol m^−2^ s^−1^ and 16/8 h light/dark photoperiod. The cultures were subcultured every five weeks. The parameters and relative spectral characteristic of the light environment are described previously [22]. These conditions have previously been found to be optimal for shoot growth and specialized metabolite production [22].

### 4.2. R. carthamoides Shoot Cultivation

A fragment of the stem with an axillary bud was taken from the shoots grown in the agar medium. This was cultured in liquid MS medium supplemented with growth regulators (given above) in a PlantForm (PlantForm AB, Hjarup, Sweden) temporary immersion system (TIS) under blue LED light (460 nm); the culture was performed for three or five weeks at PPFD of 35 μmol m^−2^ s^−1^ and 16/8 h light/dark photoperiod. The immersion time was 3 min. The shoots were immersed every 1.5, 3 or 6 h. The PlantForm bioreactor contained 500 mL of a medium delivered by air via two Hailea pumps; models ACO-9610 (power: 10 W; pressure > 0.015 Mpa; output: 10 L/min) and ACO-9602 (power: 5 W; pressure > 0.012 Mpa; output: 7.2 L/min) (Guangdong Hailea Group Co., Ltd., Chaozhou, China). More detailed information about the PlantForm bioreactor is available on the manufacturers’ website [28]. The biomass of inoculum was around 4.58 ± 0.05 g FW (0.40 ± 0.005 g DW). Each bioreactor run included 40 explants.

As the control, the shoots grown in a 300 mL stationary flask containing 50 mL of liquid medium supplemented with the same growth regulator were used. Each flask contained five explants. The biomass of inoculum was around 0.58 ± 0.04 g FW (0.05 ± 0.003 g DW). Otherwise, the culture conditions were the same as described above. The experiment consisted of three series of four flasks (*n* = 12) and three series of one PlantForm bioreactors for each conditions (*n* = 3).

In both the bioreactor and control cultures, the following parameters were recorded after three and five weeks: the multiplication rate (i.e., the mean number of buds measuring ≤0.5 cm and shoots per explant); the mean number of multiplied structures per vessel (bioreactor or flask); shoot length (cm); the percentage of buds and hyperhydricity structures; FW and DW. The DW was estimated after lyophilization using an Alpha 1–2 LDPlus freeze dryer (M. Christ Gefriertrocknungsanlagen, Osterode am Harz, Germany); the results were expressed as g/bioreactor and g/L. The experiment was repeated three times.

### 4.3. Phytochemical Analysis

The lyophilized plant material (500 mg) was powdered and extracted with 80% (*v*/*v*) aqueous methanol (i.e., an 8:2 *v*/*v* solution of methanol/water), and analyzed according to Skała et al. [23].

The CQAs and flavonoids present in the extracts were qualitatively identified using a UPLC-3000 RS system (Dionex, Dreieich, Germany) equipped with a diode-array detector and an AmaZon SL ion-trap mass spectrometer with an electrospray ionization (ESI) source (Bruker Daltonik, Bremen, Germany), as described previously [23]. UPLC separation was performed on a Kinetex XB-C18 column (1.7 μm, 150 × 2.1 mm i.d.; Phenomenex, Torrance, CA, USA). The mobile phase consisted of 0.1% formic acid in water (A) and acetonitrile containing 0.1% formic acid (B). The gradient elution program was as follows: 0–45 min, 6–26% B; 45–55 min, 26–95% B; 55–63 min, 95% B; 63–70 min, 95–6% B. The flow rate was maintained at 0.3 mL min^−1^, the column temperature at 25 °C, and the injection volume at 5 μL. UV spectra were recorded over 200–600 nm, and chromatograms were monitored at 245, 325, and 350 nm. Mass spectrometric detection was carried out in the negative ion mode under standard ion-trap conditions. The compounds were identified based on retention parameters, UV-Vis spectral characteristics, and MS^n^ fragmentation patterns, in accordance with Skała et al. [23].

Quantitative analysis was performed using a Waters 600E HPLC system equipped with a Waters 2998 PDA detector (Waters, Milford, MA, USA) as described previously [23]. Separation was achieved on a C18 Ascentis Express column (2.7 μm, 75 × 4.6 mm i.d.; Supelco, Bellefonte, PA, USA) with a C18 Ascentis Supelguard guard column (3 μm, 20 × 4 mm i.d.; Supelco, Bellefonte, PA, USA). The mobile phase consisted of 0.5% aqueous orthophosphoric acid (A) and acetonitrile (B). The gradient elution program was as follows: 0–1 min, 5% B; 1–16 min, 5–30% B; 16–17 min, 30–50% B; 17–19 min, 50% B; 19–20 min, 50–5% B; 20–25 min, 5% B. The flow rate was 1.4 mL min^−1^, the column temperature 30 °C, and the injection volume 5 μL. Detection wavelengths were selected according to the UV absorption characteristics of compound classes: 325 nm for caffeic acid derivatives, including CQAs, and 350 nm for flavonoid monoglycosides. Mono-CQA isomers were quantified as chlorogenic acid equivalents, di- and tri-CQA derivatives as cynarin equivalents, and flavonoid monoglycosides as isoquercitrin equivalents. The contents of all compounds were expressed as milligrams per gram of dry weight (mg/g DW).

### 4.4. Productivity of CQAs and Flavonoids

The productivity of CQAs and flavonoids (mg/vessel) was also calculated as the level of phenolic acids in the shoots obtained after three or five weeks from one bioreactor (or flask) from one cycle growth [42].

### 4.5. Antioxidant Activity

The aqueous methanol extract was prepared from 2.2 g of the lyophilized bioreactor-grown shoots with a 3 h immersion frequency. Briefly, the plant material was first extracted with *n*-hexane, to remove lipophilic compounds, and the resulting hexane fraction was discarded. The sample was then sonicated for 15 min in 250 mL 80% aqueous methanol, and then twice again for 15 min with 150 mL of solvent; all sonication was performed at 35 °C using an IS-20 ultrasonic bath (InterSonic, Olsztyn, Poland). The extracts were combined and evaporated to dryness to form a powder.

The antioxidant capacity of *R. carthamoides* shoots and chlorogenic acid was assessed against biologically relevant ROS in vitro using three cell-free assay systems: the hydroxyl radical (OH^•^) scavenging assay [53], in which OH^•^ radicals were generated via the Fenton reaction and detected through their reaction with salicylic acid; the hydrogen peroxide (H_2_O_2_) reduction assay [53], based on its reaction of H_2_O_2_ with 4-aminoantipyrine (4-AAP) and phenol in the presence of horseradish peroxidase; the superoxide anion (O_2_^•−^) scavenging assay [54], where the radicals were formed through xanthine oxidation and quantified by nitrotetrazolium blue chloride (NBT) reduction. Antioxidant effectiveness was expressed as EC_50_, i.e., the concentration of test plant material or compound required to reduce the initial oxidant level by 50%, calculated from dose–response (scavenging) curves. Ascorbic acid served as the reference antioxidant.

### 4.6. Statistical Analysis

The normality of the data was evaluated using the Shapiro–Wilk test. Based on the result, the data was then analysed using one-way analysis of variance (ANOVA) with Tukey’s post hoc test. Statistical significance was accepted at *p*-value < 0.05. All analyses were performed using Statistica 13.3 software (StatSoft, Krakow, Poland).

## 5. Conclusions

The use of the TIS system allows for the production of high-quality plant compounds while also being more effective and economically viable than other biotechnological methods. The shoots grown in the PlantForm bioreactor were also characterised by a higher multiplication rate, a shorter and faster shoot culture cycle compared to soil-grown plantations, which require longer cultivation times and are seasonally limited. Furthermore, in vitro culture is not affected by climatic conditions or environmental factors, making it a promising system for producing valuable specialized compounds from shoot cultures.

The Plantform TIS system described in the present study was found to be effective for rapidly propagating plant material and obtaining high-quality bioactive compounds, such as caffeoylquinic acids, from *R. carthamoides*. In addition to supporting the conservation of this endangered species, which is currently threatened by overharvesting, in vitro culture also enables the continuous acquisition of plant material, regardless of season or geographical zone (alpine and subalpine meadows and tundra).

## Figures and Tables

**Table 1:** Effect of cultivation time (3 and 5 weeks) and immersion frequency (every 1.5 h, 3 h, and 6 h) on total identified CQAs, mono-CQAs, di-CQAs, tri-CQAs, and flavonoid monoglycosides in *R. carthamoides* shoots cultured in stationary flasks and in a PlantForm bioreactor.

Culture Condition	Content	Sum of CQAs	mono-CQAs	di-CQAs	tri-CQAs	Flavonoids
Flask						
3T	mg/g DW	2.78 ± 0.15 ^d^	1.75 ± 0.10 ^c^	0.80 ± 0.03 ^b^	0.23 ± 0.02 ^cd^	0.44 ± 0.02 ^a^
	mg/flask ^1^	0.72 ± 0.04 ^E^	0.46 ± 0.02 ^F^	0.21 ± 0.008 ^E^	0.06 ± 0.006 ^C^	0.11 ± 0.004 ^F^
5T	mg/g DW	2.89 ± 0.14 ^d^	2.18 ± 0.13 ^b^	0.53 ± 0.02 ^c^	0.18 ± 0.003 ^e^	0.30 ± 0.01 ^b^
	mg/flask	1.07 ± 0.05 ^F^	0.81 ± 0.05 ^E^	0.20 ± 0.01 ^E^	0.07 ± 0.001 ^C^	0.80 ± 0.03 ^D^
PlantForm 1.5 h						
3T	mg/g DW	2.36 ± 0.06 ^e^	1.34 ± 0.03 ^e^	0.76 ± 0.01 ^b^	0.25 ± 0.02 b ^c^	0.13 ± 0.005 ^c^
	mg/bioreactor ^2^	6.02 ± 0.15 ^D^	3.43 ± 0.08 ^D^	1.95 ± 0.04 ^C^	0.65 ± 0.04 ^B^	0.34 ± 0.01 ^E^
5T	mg/g DW	2.17 ± 0.04 ^e^	1.55 ± 0.03 ^d^	0.48 ± 0.02 ^c^	0.14 ± 0.006 ^f^	0.32 ± 0.007 ^b^
	mg/bioreactor	10.13 ± 0.19 ^C^	7.23 ± 0.14 ^B^	2.25 ± 0.07 ^B^	0.65 ± 0.03 ^B^	1.52 ± 0.03 ^B^
PlantForm 3 h						
3T	mg/g DW	3.29 ± 0.10 ^c^	2.15 ± 0.04 ^b^	0.81 ± 0.03 ^b^	0.33 ± 0.03 ^a^	0.34 ± 0.01 ^b^
	mg/bioreactor	6.70 ± 0.20 ^D^	4.37 ± 0.09 ^C^	1.65 ± 0.07 ^D^	0.67 ± 0.06 ^B^	0.69 ± 0.02 ^D^
5T	mg/g DW	4.46 ± 0.24 ^a^	3.27 ± 0.14 ^a^	1.00 ± 0.07 ^a^	0.18 ± 0.03 ^def^	0.47 ± 0.03 ^a^
	mg/bioreactor	16.13 ± 0.85 ^A^	11.85 ± 0.49 ^A^	3.63 ± 0.27 ^A^	0.65 ± 0.09 ^B^	1.70 ± 0.12 ^A^
PlantForm 6 h						
3T	mg/g DW	2.27 ± 0.08 ^e^	1.27 ± 0.03 ^e^	0.71 ± 0.03 ^b^	0.28 ± 0.02 ^ab^	0.43 ± 0.02 ^a^
	mg/bioreactor	5.97 ± 0.20 ^D^	3.35 ± 0.09 ^D^	1.88 ± 0.07 ^CD^	0.75 ± 0.04 ^B^	1.14 ± 0.05 ^C^
5T	mg/g DW	3.75 ± 0.10 ^b^	2.39 ± 0.05 ^b^	1.04 ± 0.03 ^a^	0.31 ± 0.02 ^ab^	0.42 ± 0.01 ^a^
	mg/bioreactor	12.56 ± 0.33 ^B^	8.01 ± 0.17 ^B^	3.49 ± 0.11 ^A^	1.05 ± 0.08 ^A^	1.40 ± 0.04 ^B^

^1,2^—the content of CQAs present in mg per vessel (one flask or one bioreactor) in single cycle of growth. The results are mean values ± SD. There is no difference (*p* < 0.05) among the means for the same metabolite content marked with the same letter within the columns. Small letters are for metabolite content in mg/g dry weight (DW), and big letters are for metabolite content in mg/flask or bioreactor. 3T—3 weeks of growth; 5T—5 weeks of growth; mono-CQAs—mono-caffeoylquinic acid derivatives; di-CQAs—di-caffeoylquinic acid derivatives; tri-CQAs—tri-caffeoylquinic acid derivatives.

**Table 2:** Effect of cultivation time (3 and 5 weeks) and immersion frequency (every 1.5 h, 3 h, and 6 h) on *R. carthamoides* shoot length, ratio of shoots to buds, and hyperhydricity structures (%).

Culture Condition	Shoot Length	Ratio of Shoots to Buds (%)	Hyperhydricity Structures (%)
Flask			
3T	2.46 ± 0.09 ^b^	71.9:28.1	6.1
5T	3.28 ± 0.07 ^a^	99.6:0.4	3.8
PlantForm 1.5 h			
3T	1.27 ± 0.02 ^e^	75.7:24.3	13.0
5T	2.37 ± 0.07 ^b^	90.2:9.8	4.4
PlantForm 3 h			
3T	1.49 ± 0.03 ^d^	74.5:25.5	6.3
5T	2.46 ± 0.04 ^b^	97.7:2.3	4.1
PlantForm 6 h			
3T	1.96 ± 0.04 ^c^	94.7:5.3	4.9
5T	2.06 ± 0.03 ^c^	99.8:0.2	9.6

The results are mean values ± SE. Means marked with the same letter indicate no significant difference (*p* < 0.05). 3T—3 weeks of growth; 5T—5 weeks of growth.

## Data Availability

Data are contained within this article.

## Abbreviations

The following abbreviations are used in this manuscript:

TIS	Temporary immersion system
CQA	Caffeoylquinic acid derivatives
